# Medical Utilization and Expenditures Before and After Total Knee and Hip Arthroplasty in Osteoarthritis in Seoul, South Korea: A Retrospective Matched Cohort Study

**DOI:** 10.3390/healthcare14193186

**Published:** 2026-09-26

**Authors:** Yejin Kim, Hyuk-Jae Chang, Tae Hyun Kim

**Affiliations:** 1 Department of Public Health & AI, Graduate School of Cancer Science and Policy, National Cancer Center, Goyang 10408, Republic of Korea; yejin.k75@gmail.com; 2 Division of Cardiology, Severance Hospital, Seoul 03722, Republic of Korea; 3 Department of Biohealth Industry Management, Graduate School of Transdisciplinary Health Sciences, Yonsei University, Seoul 03722, Republic of Korea

**Keywords:** total knee arthroplasty, total hip arthroplasty, osteoarthritis, medical utilization, medical expenditures, National Health Insurance, chronic disease management

## Abstract

**Background:** Osteoarthritis (OA) is a chronic disease requiring long-term management, and its burden is expected to grow with population aging, including in South Korea’s rapidly aging population. Although total knee arthroplasty (TKA) and total hip arthroplasty (THA) are common interventions for OA, their long-term association with healthcare utilization and expenditures across benefit classifications remains unclear. This study examined differences in medical utilization and expenditures, disaggregated by benefit classification, between patients who underwent TKA/THA and matched non-surgical controls, before and after surgery. **Methods:** This retrospective cohort study used 1:3 direct matching on sex, calendar date, and OA duration to compare patients who underwent TKA/THA (2012–2019) with matched non-surgical controls at a tertiary hospital in Seoul, South Korea. Outcomes were inpatient and outpatient medical utilization and expenditures over 30-day intervals, disaggregated by benefit classification (covered, uncovered, and out-of-pocket payments (OOPs)). Generalized estimating equation models with an autoregressive correlation structure estimated associations before and after surgery. Poisson regression was used for inpatient and outpatient visits, and Negative Binomial regression was used for LOS given evidence of overdispersion and excess zeros. Medical expenditures were log-transformed and analyzed using normal distribution models. **Results:** A total of 351 surgical patients and 984 matched controls were included. Before surgery, the surgical cohort had lower inpatient utilization and expenditures than matched controls. Inpatient visits were 27% fewer (95% CI: 0.62–0.87). Covered, uncovered, and OOP expenditures were 25% (95% CI: 0.65–0.86), 24% (95% CI: 0.67–0.87), and 25% (95% CI: 0.65–0.86) lower, respectively. Outpatient OOPs were 46% higher (95% CI: 1.30–1.64). After surgery, this pattern reversed for inpatient services. Visits (exp(β) = 2.09, 95% CI: 1.64–2.67), average length of stay (exp(β) = 2.51, 95% CI: 1.78–3.54), covered expenditures (exp(β) = 3.12, 95% CI: 2.51–3.89), uncovered expenditures (exp(β) = 2.93, 95% CI: 2.41–3.58), and OOPs (exp(β) = 2.99, 95% CI: 2.42–3.71) were all higher than matched controls. Outpatient visits (exp(β) = 0.74, 95% CI: 0.65–0.84), covered expenditures (exp(β) = 0.52, 95% CI: 0.38–0.70), uncovered expenditures (exp(β) = 0.37, 95% CI: 0.24–0.58), and OOPs (exp(β) = 0.57, 95% CI: 0.48–0.68) were all lower. These results were consistent after Bonferroni correction for multiple comparisons (α = 0.0028). **Conclusions:** The association between arthroplasty and healthcare utilization differed markedly by period. Inpatient burden concentrated around surgery, while outpatient utilization at this institution declined thereafter. This may be partly explained by post-surgical care being dispersed toward non-tertiary institutions. These findings are hypothesis-generating and should be examined further using multi-institutional data. If substantiated, resource planning may need to prioritize the perioperative period while strengthening community-level coverage during recovery.

## 1. Introduction

Knee and hip osteoarthritis (OA) is a chronic, progressive degenerative disease that requires long-term management, serving as a major cause of disability in adults over 60 years of age worldwide and resulting in significant pain, functional limitations, and reduced quality of life for patients [1,2]. This burden is particularly relevant to South Korea, where the population is aging rapidly. As of 2025, 21% of the national population and 20% of Seoul’s population were aged 65 or older, both exceeding the 20% threshold that defines a “super-aged society” [3]. As South Korea’s older population continues to grow, the burden of OA-related disability described above is likely to become more pronounced. This underscores the importance of sustainable, long-term management of this chronic disease.

OA is closely linked to aging, with age recognized as the most prominent risk factor for its onset and progression [4,5]. Within the long-term management of OA, Total Knee Arthroplasty (TKA) and Total Hip Arthroplasty (THA) represent a critical intervention point, widely recognized as clinically effective and cost-effective treatments for pain relief and functional improvement [6,7,8]. These surgeries provide sustained effects of pain reduction and quality of life improvement for up to 5 years postoperatively [6]. However, whether patients in need ultimately undergo TKA/THA is not determined by clinical need alone. Insurance coverage, out-of-pocket payment (OOP) burden, and other socioeconomic factors have also been shown to influence this [9]. Surgery itself also does not conclude the management of OA as a chronic condition; rather, it marks a transition to a new phase of healthcare utilization that continues to require monitoring, follow-up, and resource allocation over the long term [10].

The demand for joint replacement surgery is rapidly increasing worldwide [11,12]. In the US, TKA is projected to increase by 401% and THA by 284% by 2040, with substantial increases also expected in the UK by 2035 [13,14]. In South Korea, the number of TKA procedures performed continuously increased during the period from 2011 to 2018, with particularly marked increases observed in patients aged 70 years and older [15]. This growing demand highlights the importance of understanding the long-term healthcare utilization and expenditure patterns associated with surgical intervention, in order to support sustainable resource allocation for patients with chronic musculoskeletal disease.

As demand for joint replacement surgery increases, optimizing medical resource utilization and minimizing costs while enhancing patient safety and satisfaction becomes essential [11]. Previous studies have primarily focused on analyzing medical costs following TKA or THA surgery in Medicare patients in the US or on the cost-effectiveness of specific technologies [16,17,18,19]. Comparatively limited attention has been paid to long-term, longitudinal comparisons against non-surgical controls or to how utilization patterns differ across benefit classification.

South Korea’s National Health Insurance (NHI) operates as a single, government-run insurance system covering the entire population, under which medical expenditures are classified according to benefit status: NHI-covered services and uncovered services. Total medical expenditures therefore consist of NHI-covered expenditures (financed through NHI) and uncovered expenditures (paid entirely by the patient), while OOPs represent the patient’s actual financial burden, combining copayments for covered services and the full cost of uncovered services. How this composition shifts following joint replacement surgery—that is, whether increases are driven by NHI-financed spending, patient copayments, or uncovered service use—may inform policy discussions on the sustainability of NHI financing and the adequacy of coverage for chronic musculoskeletal disease management.

Therefore, this study aims to compare medical utilization and expenditures between patients who underwent TKA/THA and matched non-surgical controls over a long-term follow-up period. By examining differences in healthcare utilization and cost composition, by benefit classification, between the surgical and matched controls, both before and after surgery, this study seeks to provide evidence relevant to the sustainable, patient-centered management of chronic musculoskeletal disease.

## 2. Methods

### 2.1. Data Source and Study Subjects

This study utilized data from a longitudinal cohort of patients with degenerative chronic disease (OA, chronic kidney disease (CKD), dementia, and chronic obstructive pulmonary disease (COPD)) at Severance Hospital in Seoul, South Korea, from 2006 to 2022, reflecting the period from which the hospital’s electronic medical record system became fully established. Patients were tracked using a unique patient identifier, distinct from visit-level claim numbers, ensuring consistent identification across encounters. Visit records with negative length of stay (LOS < 0), occurring during an inpatient stay with zero recorded expenditure, were treated as data errors and excluded prior to cohort construction. OA was defined using the following International Classification of Diseases, 10th Revision codes: chronic postrheumatic arthropathy of the pelvis, thigh, and lower leg (M12.05, M12.06), palindromic rheumatism of the pelvis, thigh, and lower leg (M12.35, M12.36), polyarthrosis (M15), coxarthrosis (M16), and gonarthrosis (M17) [20]. The study population included patients aged 60 years or older at the time of initial OA diagnosis.

The nationwide implementation of specialized hospital systems for OA in 2011, which potentially influenced healthcare utilization patterns among OA patients [21]. Accordingly, the surgical cohort was defined as patients who underwent TKA or THA from 2012 onward, allowing utilization patterns to stabilize following this policy change. Non-surgical controls were defined as patients with no TKA/THA claims recorded at any point during the entire study period (2006–2022). Using the surgery date as the index date for the surgical group, these controls were matched to surgical patients using direct matching on sex, calendar date, and OA duration. OA duration was pre-calculated for every recorded visit as the interval since each patient’s first OA diagnosis. A risk-set sampling approach was used, in which eligible controls were restricted to non-surgical OA patients with a recorded visit on the same calendar date as the corresponding surgical patient’s index date. Controls were matched exactly on sex, calendar date, and OA duration, and by nearest neighbor on age among the resulting candidates. Up to three controls were selected per surgical patient, matched sequentially without replacement, such that each control could be selected for only one surgical patient. No caliper was applied to the age difference. Each matched control was assigned the same calendar-time index date as its corresponding surgical patient. Time points were calculated in 30-day intervals from the index date, and patients with fewer than 36 pre-index and 36 post-index 30-day time points were then excluded.

### 2.2. Measures

The primary outcome variables were average per-person medical utilization and expenditures over 30-day periods. Medical utilization measures included the number of inpatient visits, average LOS, and number of outpatient visits, each calculated as the mean value per patient within each 30-day interval. Average LOS included zeros for patients without an inpatient visit during a given interval. Medical expenditures were categorized into inpatient and outpatient services, with further subdivision into total medical expenditures, NHI payments for covered services, payments for uncovered services, and OOPs. OOPs represented the sum of patient copayments for covered services and the full cost of uncovered services, since uncovered services were not reimbursed by NHI and were paid entirely by the patient. All 351 surgical patients and 984 matched controls contributed data at every 30-day time point. If there was no recorded utilization at the time points, the utilization and expenditures were coded as zero rather than treated as missing or censored. All medical expenditures were adjusted for inflation using the 2020 healthcare consumer price index and converted to US dollars based on annual exchange rates [22,23].

The primary exposure variable was TKA/THA status, a time-varying indicator of whether a patient had undergone surgery, identified through claims for “total hip and knee joint (complex) arthroplasty.” Patients were classified as untreated prior to surgery and throughout follow-up for matched controls, and as treated from the date of surgery onward.

Covariates included patient demographics (sex, age), insurance status (NHI/Medical Benefit/Other), duration of OA, Charlson Comorbidity Index (CCI) scores [24], and comorbid conditions including CKD, dementia, and COPD. OA duration was calculated for every recorded visit of every patient, in 30-day units, as the gap between that visit date and the date of first OA diagnosis recorded at the study hospital. Because covariates were drawn from a broader claims database encompassing multiple chronic conditions, OA status was included as a time-varying covariate to account for the fact that, particularly for time points preceding the index date, some matched controls had not yet received an OA diagnosis; OA status was coded as “Yes” from the time of diagnosis onward. Each comorbid condition was similarly coded as “Yes” from the time of diagnosis onward. Receipt of revision arthroplasty (TKA/THA re-surgery) was included as a time-varying covariate, coded as “Yes” from the time of the revision procedure onward, to distinguish its effect from that of the primary surgical exposure. Time point and year fixed effects were included to control for temporal trends and secular changes in medical utilization patterns.

### 2.3. Analysis

Baseline characteristics of the surgical and matched cohorts were assessed at time zero. For categorical variables, chi-square tests were performed, while continuous variables were analyzed using independent-samples t-tests and Wilcoxon rank-sum tests as appropriate. Additionally, balance in baseline covariates between surgical and matched cohorts was assessed using standardized mean differences (SMD).

To examine temporal patterns in medical utilization, we calculated mean medical utilization and total medical expenditure at 1, 2, and 3 years before and after time zero. Medical expenditures over time were visualized using cumulative graphs to illustrate spending trends across the study period.

To assess the longitudinal association between arthroplasty and medical utilization and expenditure measures while adjusting for covariates, GEE models were fitted with the following main-effects equation:

*g*(*E*[*Y_it_*]) = *β*_0_ + *β*_1_∙Treat*_i_* + *β*_2_∙Surgery*_it_* + *β*_3_∙Time*_t_* + *γ*^T^∙Year_t_ + *β*^T^∙X*_it_*

Treat*_i_* is a time-invariant indicator coded as 1 for surgical patients and 0 for matched controls; Surgery*_it_* is a time-varying indicator coded as 1 from the date of surgery onward for surgical patients and equal to 0 at all time points for matched controls, since controls did not undergo surgery; Time*_t_* is a linear time term; Year_t_ is a vector of year indicator (dummy) variables representing year fixed effects, with corresponding coefficient vector *γ*; and X*_it_* denotes the remaining covariates. A cohort-by-time interaction term was not included, as the timing of surgery reflected patients’ own clinical decisions rather than an external assignment mechanism, and results are interpreted as associations rather than causal treatment effects.

Under this equation, the estimated difference between the surgical and matched cohorts is *β*_1_ before surgery and *β*_1_ + *β*_2_ after surgery. We refer to the latter as the post-surgery estimate; it is obtained as the sum of the surgical cohort and post-surgery indicator coefficients, with standard errors derived from the model’s covariance matrix. For matched cohorts, both Treat*_i_* and Surgery*_it_* remain 0 throughout follow-up. As a result, this model does not estimate any change in outcomes among controls around their assigned index date. Instead, it estimates the magnitude of the surgical-control difference separately for the periods before and after surgery, using each control’s assigned index date to define the corresponding comparison time points.

A continuous cohort-by-time interaction was also not included. Unlike a continuous interaction, which would impose a single linear trend in the surgical-control difference across the entire follow-up period, the timing of surgery is inherently a discrete event rather than a gradual process. The binary pre/post distinction, captured by the Surgery indicator described above, was therefore considered more appropriate than a continuous linear interaction.

Considering working correlation structures, an autoregressive correlation structure was specified based on the quasi-likelihood under the independence model criterion (QIC) to account for within-subject correlation over time. For medical utilization outcomes, Poisson regression with a log link was used, while medical expenditures were log-transformed and analyzed using a normal distribution with an identity link. Overdispersion and excess zeros were assessed for each utilization outcome, based on the Pearson chi-square/df ratio and the observed versus expected proportion of zeros under a Poisson distribution. Overdispersion was minimal for both inpatient and outpatient visits, and the observed proportion of zeros did not exceed the expected proportion for either outcome. However, for LOS, both substantial overdispersion and excess zeros were identified; a Negative Binomial GEE model was therefore used. Exponentiated coefficients for each utilization outcome are interpreted as rate ratios, and those for each expenditure outcome are interpreted as ratios of geometric means, for the surgical relative to the matched control cohort. Because medical expenditures included zero values for time points without recorded utilization, a small constant (0.0001) was added to all expenditure values prior to log transformation. The association during the post-surgery period was estimated as the sum of the surgical cohort and post-surgery indicator coefficients, with standard errors derived from the model’s covariance matrix.

All statistical analyses were performed using SAS version 9.4 (SAS Institute, Cary, NC, USA), and statistical significance was set at *p* < 0.05. Given the number of outcomes examined (nine utilization and expenditure measures, each assessed separately before and after surgery, for a total of 18 estimates), a Bonferroni correction was applied to account for multiple comparisons. The adjusted significance threshold was α = 0.0028 (≈0.05/18).

## 3. Results

Direct matching resulted in 1770 surgical patients matched to 3117 controls. After excluding patients with fewer than 36 pre-index and 36 post-index 30-day time points, the final sample comprised 351 surgical patients and 984 matched controls (see Figure 1).

Table 1 presents the baseline characteristics of the surgical and matched cohorts at time zero. Both cohorts had a mean age of 71 years, with the majority being female (87.5% surgical vs. 84.3% matched), showing no statistically significant difference between groups. While most participants in both cohorts held NHI coverage (surgical: 98.9%, matched: 94.7%), there was a statistically significant difference between the two groups (*p* = 0.0039). This discrepancy between the SMD (0.24) and the *p*-value likely reflects the chi-square test’s sensitivity to sample size, given the relatively large combined sample (*n* = 1335). Regarding OA duration, the surgical cohort underwent surgery at a mean of 7.5 months after initial diagnosis, while the matched controls had a mean osteoarthritis duration of 4.4 months at time zero, representing a statistically significant difference (*p* < 0.0001). At time zero, CCI scores were slightly higher in the matched controls (2.6 points), though this difference was not statistically significant between cohorts. The prevalence of comorbid conditions was low in both groups, with most participants free from CKD, dementia, and COPD, with no statistically significant differences observed between groups.

Table 2 and Table 3 present mean medical utilization and total medical expenditures at 30-day intervals for 12, 24, and 36 months (approximately 1, 2, and 3 years) before and after time zero. Outside of time zero, medical utilization and expenditure levels were generally low and broadly comparable between the two cohorts; however, both cohorts showed a marked, transient change at time zero itself.

Both groups demonstrated low mean numbers of inpatient visits per 30-day period (surgical: 0.03–0.11 visits, matched: 0.06–0.07 visits) and short LOS (surgical: 0.20–0.47 days, matched: 0.15–0.33 days). Outpatient visit frequencies were also comparable, with fewer than two visits per month for both cohorts (surgical: 1.56–1.89 visits, matched: 1.72–2.00 visits). However, at time zero, the surgical cohort showed an increase in inpatient utilization, with 1.03 inpatient visits and 5.17 days’ length of stay, substantially higher than both pre- and post-surgical periods and compared to the matched controls. Conversely, outpatient visits decreased to 0.98 in the surgical cohort while increasing to 2.29 in the matched controls at time zero.

Medical expenditures followed similar patterns. Inpatient expenditures were generally, though not consistently, higher in the surgical cohort than the matched controls (surgical: $127.0–$329.1, matched: $107.8–$223.4); this pattern reversed at +24 months, when inpatient expenditure was lower in the surgical cohort ($127.0) than in matched controls ($223.4). Outpatient expenditures were lower in the surgical cohort throughout (surgical: $101.8–$156.8, matched: $138.0–$172.2). At time zero, inpatient expenditures increased dramatically in the surgical cohort, reaching a mean of $7102.2, while the matched controls also showed an increase to $526.0 compared to other time periods. In contrast, outpatient expenditures at time zero decreased substantially in the surgical cohort to $41.9, while the matched controls showed a modest decrease to $131.3.

Figure 2 illustrates the cumulative inpatient and outpatient expenditures over time for both cohorts. For inpatient expenditures, the two cohorts showed comparable accumulation up to approximately the 24th 30-day interval before time zero (approximately 24 months); thereafter, the surgical cohort’s cumulative expenditures began rising at a steeper rate than the matched controls, followed by a sharp discontinuity at time zero reflecting the immediate costs associated with the TKA or THA procedure. Following this acute increase, the surgical cohort’s cumulative expenditures rose faster than the matched controls throughout the remaining follow-up period, resulting in a widening gap between the two cohorts by the end of the three-year observation window. In contrast, outpatient expenditures showed more consistent linear accumulation patterns in both groups, with the matched controls maintaining a slightly higher rate of accumulation than the surgical cohort throughout the observation window.

Table 4 presents the estimated GEE coefficients for the before-and-after surgery comparison between the surgical and matched controls. Before the surgery, the surgical cohort had 27% fewer inpatient visits (95% CI: 0.62–0.87, *p* = 0.0003) than matched controls, while LOS and outpatient visits did not differ significantly. Inpatient expenditures were also lower in the surgical cohort: covered expenditures were 25% lower (95% CI: 0.65–0.86, *p* < 0.0001), uncovered service expenditures were 24% lower (95% CI: 0.67–0.87, *p* < 0.0001), and OOPs were 25% lower (95% CI: 0.65–0.86, *p* < 0.0001). For outpatient services, OOPs were 46% higher (95% CI: 1.30–1.64, *p* < 0.0001); covered and uncovered expenditures did not differ significantly.

In the resulting after surgery comparison, the surgical cohort had more inpatient visits (exp(β) = 2.09, 95% CI: 1.64–2.67, *p* < 0.0001) and LOS (exp(β) = 2.51, 95% CI: 1.78–3.54, *p* < 0.0001) than matched controls; covered expenditures (exp(β) = 3.12, 95% CI: 2.51–3.89, *p* < 0.0001), uncovered expenditures (exp(β) = 2.93, 95% CI: 2.41–3.58, *p* < 0.0001), and OOPs (exp(β) = 2.99, 95% CI: 2.42–3.71, *p* < 0.0001) were also higher. For outpatient services, visits were lower (exp(β) = 0.74, 95% CI: 0.65–0.84, *p* < 0.0001), and covered expenditures (exp(β) = 0.52, 95% CI: 0.38–0.70, *p* < 0.0001), uncovered expenditures (exp(β) = 0.37, 95% CI: 0.24–0.58, *p* < 0.0001), and OOPs were lower (exp(β) = 0.57, 95% CI: 0.48–0.68, *p* < 0.0001) than matched controls. These results were consistent after Bonferroni correction for multiple comparisons.

## 4. Discussion

This study longitudinally examined medical utilization and expenditures between a TKA or THA surgical cohort and a propensity score-matched non-surgical cohort of OA patients. Prior to surgery, the surgical cohort was associated with lower inpatient utilization and expenditures relative to matched controls, alongside higher outpatient OOPs. Following surgery, this pattern reversed for inpatient services: the surgical cohort showed substantially higher inpatient utilization and expenditures than matched controls, while outpatient utilization and expenditures, including OOPs, were lower. These findings indicate that the association between arthroplasty and healthcare utilization differs markedly by period. Inpatient burden rose sharply around the time of surgery—likely reflecting the acute perioperative period rather than a sustained long-term difference—while outpatient burden recorded at this single tertiary hospital declined in the post-surgery period.

This reversal could reflect the acute costs of the procedure itself, or greater disease severity among patients approaching surgery. More severe cases are both more likely to require inpatient care and more likely to proceed to surgery [25]. Bozic et al. [26] reported that although outpatient visits declined slightly in the year following total joint arthroplasty compared to the presurgical period, the proportion of patients hospitalized increased, and total costs during follow-up were 18% higher than before surgery, driven primarily by hospital readmission-related inpatient costs rather than outpatient care, which was in fact slightly lower after surgery. This pattern also resembles findings from studies comparing only pre- and post-surgical periods with patients who underwent surgery; expenditures increased following surgery despite reductions in LOS [20,27]. The present study’s within-cohort comparison captures the change experienced by surgical patients themselves, independent of the matched controls. This comparison also showed a marked increase in inpatient visits (exp(β) = 2.85, 95% CI: 2.38–3.42) and LOS (exp(β) = 2.83, 95% CI: 2.19–3.65) following surgery. This is consistent with the interpretation that the rise in inpatient burden likely reflects the acute costs of the procedure itself, rather than differences in overall care-seeking behavior between the two cohorts. However, this within-cohort comparison does not explain the pre-surgery difference between the surgical and control cohorts noted above, which may instead reflect residual confounding by indication.

The increase in inpatient utilization and expenditure around the surgery may be further amplified among older surgical patients, who often present with greater frailty and comorbidity, requiring more intensive perioperative care [16]. This may be particularly relevant to the patient population in this study, given South Korea’s tiered healthcare delivery system. Prior research has shown that hospital density is one of the strongest correlates of elderly population concentration across districts in Seoul [28]. The study institution is one such tertiary hospital, where reimbursement rates and associated costs are structurally higher than at primary or secondary facilities. This may partly explain the substantial increase in inpatient expenditures observed around the time of surgery. Conversely, given the wide availability of primary, secondary, and other lower-tier healthcare institutions in Seoul, patients recovering from surgery may have redirected their outpatient care to more accessible, lower-cost facilities outside the tertiary setting. This offers one possible explanation, among others, for the decline in outpatient utilization observed at this institution.

The observed decline in post-surgical outpatient utilization at this institution may partly reflect broader patterns in how Korean OA patients seek care following surgery, rather than a true reduction in overall outpatient need. Jeon et al. [29] tracked healthcare utilization and expenditures among musculoskeletal surgical patients following hospital discharge and found that, while conventional (Western medicine) outpatient visits declined after surgery, visits to Korean medicine clinics—predominantly at the primary and secondary care level—increased from around 6 months post-surgery onward. This may be explained, in part, by patients—particularly during the stabilization and rehabilitation phase—increasingly seeking pain management and functional recovery through a range of care options outside the institution where surgery was performed [30,31]. This suggests that outpatient care following surgery may be, in part, dispersed toward non-tertiary institutions. However, because the present study did not include data from Korean medicine clinics or other non-tertiary facilities, this explanation remains speculative and should be considered a hypothesis requiring confirmation with more comprehensive data. Nonetheless, this hypothesis is broadly consistent with recent Korean health policy directions. Legislation enacted in 2024 mandates home-based rehabilitation, multidisciplinary support, and financial protection for high-risk surgical patients [32], marking a systematic shift from institution-centered to community-centered recovery pathways. If this policy framework becomes more established, the pattern observed in the present study—reduced outpatient utilization at the tertiary hospital following surgery—could become even more pronounced, a possibility that future research should be examined.

The pronounced but transient rise in inpatient burden around the time of surgery, together with the observed decline in post-surgical outpatient utilization at this tertiary hospital, illustrates an important pattern. Healthcare needs for patients undergoing joint replacement surgery may shift markedly across distinct phases of care. Disaggregating medical utilization and expenditures by period and by benefit classification may offer information relevant to identifying where chronic disease management resources are concentrated across the surgical care continuum.

In this cohort, the majority of participants were female, consistent with the well-established higher prevalence of knee and hip osteoarthritis among women compared to men, particularly after the age of 50 [1,2,33]. This sex disparity has been attributed to a combination of hormonal, genetic, and anatomical factors, although the relative contribution of each remains debated [33]. Because OA prevalence itself differs by sex, this predominance reflects the underlying disease population at this institution rather than a selection artifact specific to the surgical or matching procedures.

### Limitations

This study has several limitations. Because claims data from a single tertiary hospital were used, information about healthcare utilization at other healthcare institutions was not included. Therefore, the total healthcare utilization and medical expenditures of OA and TKA/THA patients may have been underestimated. Particularly, the previously mentioned specialized joint hospital system has increased the likelihood of patients using multiple healthcare institutions, and the possibility that these changes influenced the study results cannot be excluded [21]. In addition, due to the characteristics of claims data collected under the fee-for-service system, it was difficult to accurately distinguish medical expenditures that could be directly attributed to OA and TKA/THA. Therefore, it is unclear whether the increased outpatient OOPs observed in the surgical cohort are mainly related to OA or surgery itself, or are due to management of other comorbidities.

Requiring at least three years of observed data before and after the index date may have introduced selection bias. Patients who died, transferred care, or otherwise discontinued visits within this window could not be included. This requirement was necessary because, within a single-institution database, the absence of subsequent visits cannot be reliably distinguished from death, transfer of care, or simple discontinuation. This may limit the generalizability of our findings to patients who survived and continued to receive care at the study institution.

Although propensity score matching is a widely used approach for this type of comparative cohort study, it could not be implemented within the air-gapped network environment used for this study. Direct matching on sex, calendar date, and OA duration was used instead, with nearest-neighbor matching on age. This approach does not provide the multivariable balance achieved by propensity score methods, and no caliper was applied to restrict the maximum age difference between matched pairs. Age was nonetheless well balanced between the two cohorts in the matched sample (SMD = 0.08). Furthermore, direct matching has been noted to be advantageous when the number of confounders to be balanced is small, as it allows transparent identification of exactly balanced pairs and avoids potential bias from propensity score model misspecification [34]. Insurance status and CCI were not included as matching variables, as incorporating additional matching criteria substantially reduced the number of eligible controls within this single-institution cohort. This likely contributed to the residual imbalance observed for these variables (SMD = 0.24 and 0.11, respectively). Although OA duration was used as a matching variable, a residual imbalance nonetheless remained (SMD = 0.24). This may reflect the coarseness of OA duration as a matching criterion: because OA duration was calculated in 30-day units, patients matched on the same interval value could still differ in actual duration by up to 29 days, and such differences may accumulate into a between-group difference at the aggregate level. However, an SMD below 0.25 is recognized as a reasonable and acceptable threshold for concluding that the distribution of covariates is relatively well-balanced [35]. We also employed a double-adjustment strategy by including OA duration as a covariate in the GEE models. This approach may reduce, though may not fully eliminate, confounding arising from the residual imbalance [35]. Additionally, because OA duration was defined based on the earliest diagnosis recorded at the study hospital rather than actual symptom onset, it may underestimate the true clinical duration of OA prior to arthroplasty.

Clinical factors such as patient severity, functional status, obesity, and duration of pre-surgical symptoms may not have been adequately controlled. Because this study focused only on medical utilization and expenditures, outcome indicators such as clinical effectiveness of surgery, patient satisfaction, and quality of life improvement were not evaluated.

As the decision to undergo arthroplasty is related to disease severity and other clinical factors not fully captured, residual confounding by indication remains a concern. The overall post-surgery estimates may also be strongly influenced by the index surgical episode itself. Because the post-surgery estimate reflects an average across all post-index time points, this acute perioperative increase may disproportionately drive the overall estimate. These findings should therefore be interpreted with caution when generalizing to longer-term post-surgery patterns. TKA and THA were pooled into a single surgical cohort for analysis. Of the 351 surgical patients, 317 (90.3%) underwent TKA and 34 (9.7%) underwent THA. This pooling was adopted primarily due to the limited number of THA cases, which precluded a statistically meaningful separate analysis for this subgroup. However, TKA and THA may differ in recovery trajectories and healthcare utilization patterns, and this pooling may obscure procedure-specific differences.

## 5. Conclusions

This study found that the association between arthroplasty and medical utilization differed markedly by period. There was a substantial rise in inpatient burden concentrated around the acute perioperative period, alongside a decline in outpatient utilization and OOPs recorded at this institution. This decline may be partly explained by post-surgical care being dispersed toward non-tertiary institutions. These findings are hypothesis-generating and should be examined further using multi-institutional data and clinical outcome indicators to clarify their implications for the value of joint replacement surgery. Future research incorporating clinical outcome indicators and, potentially, formal cost-optimization frameworks could help translate these descriptive patterns into actionable resource planning. If these patterns are substantiated, resource planning for joint replacement surgery may need to prioritize the perioperative period while ensuring adequate coverage and care coordination at the primary and secondary care level to support patients through recovery.

## Figures and Tables

**Figure 1:**
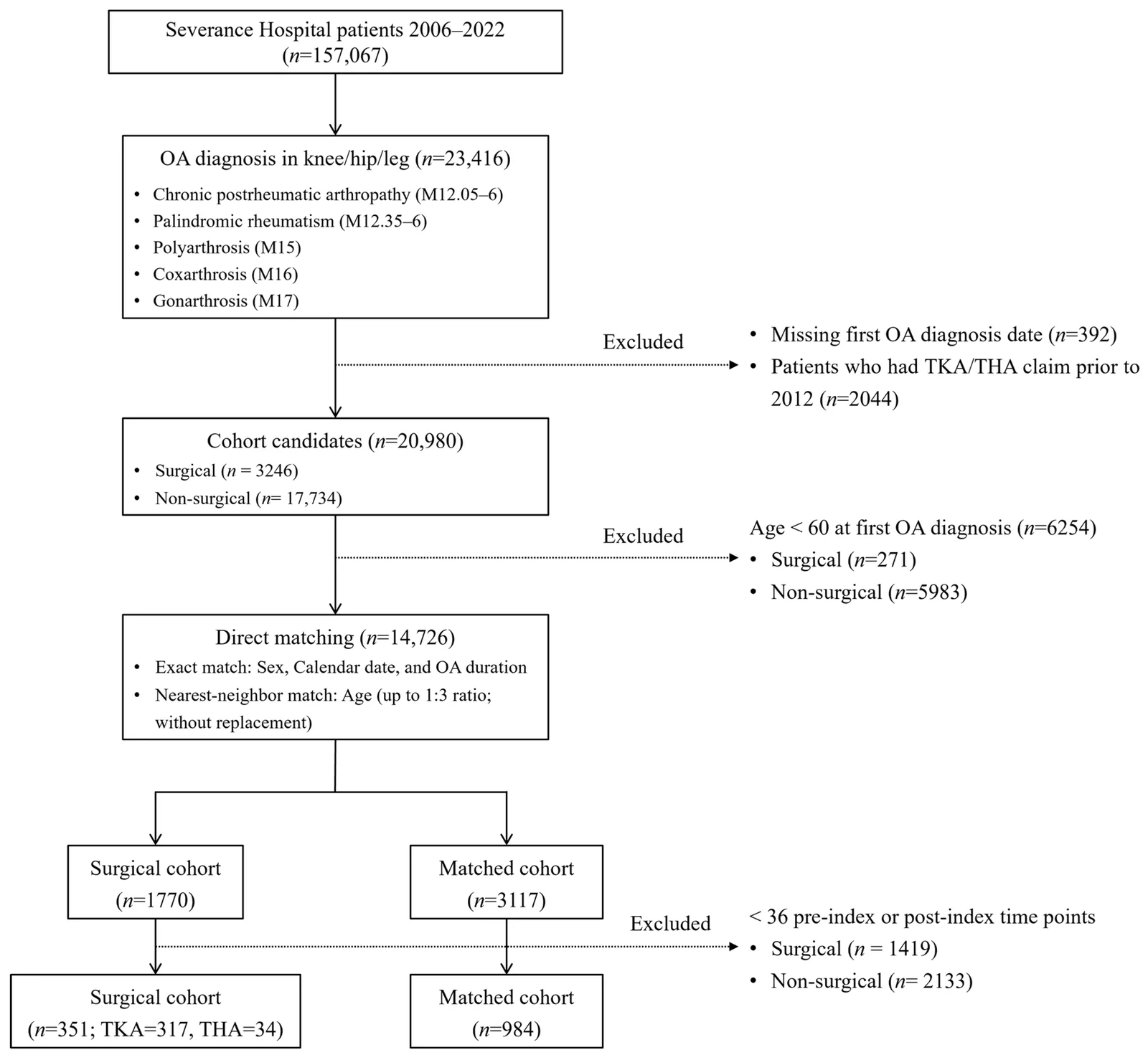
Study flow chart. Legend: Figure 1 is the flow chart of study participant selection. *N* is the number of patients.

**Figure 2:**
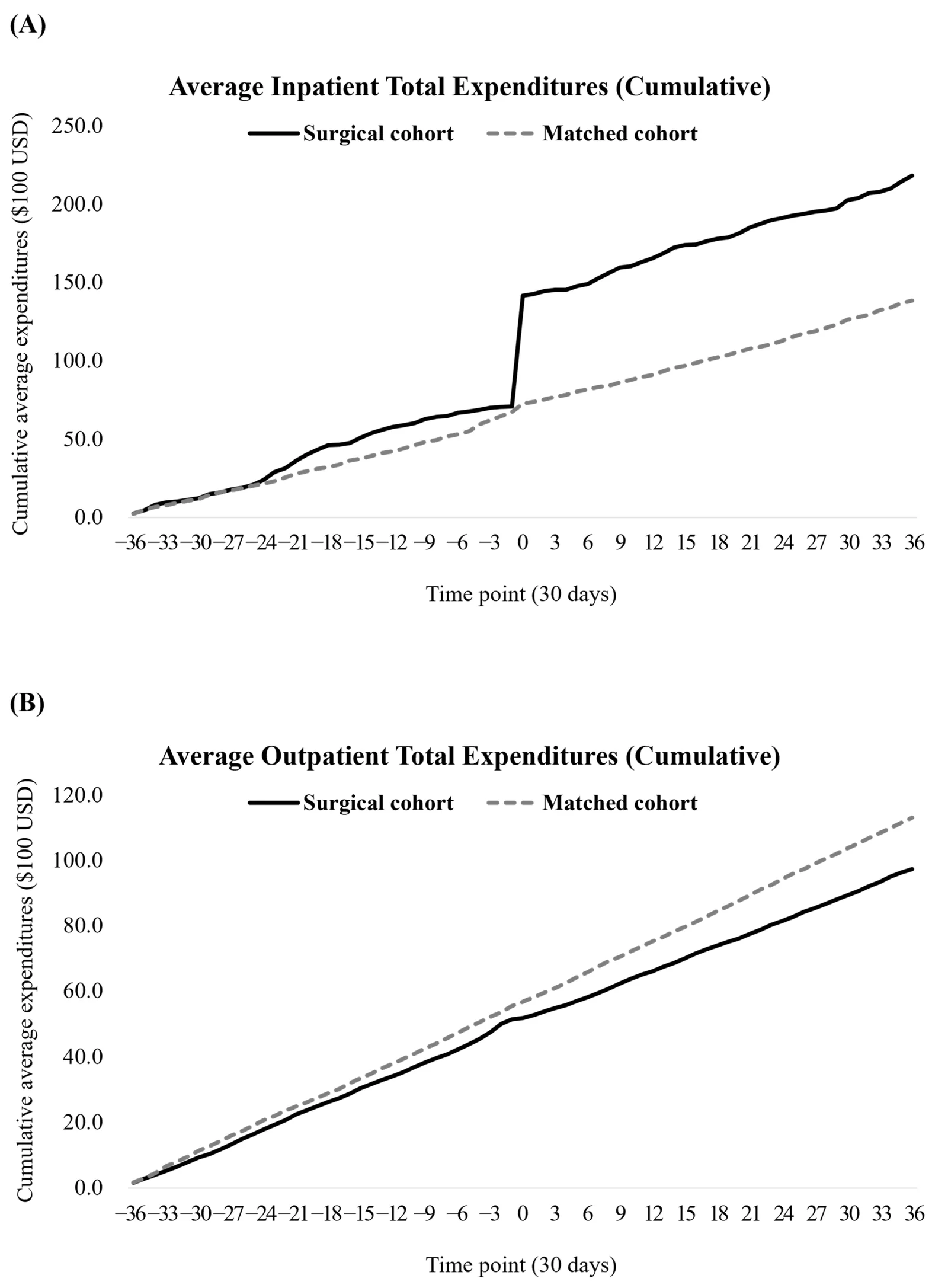
Cumulative inpatient and outpatient total expenditures over time. Legend: Figure 2 shows the cumulative average total medical expenditures by time point for inpatient (**A**) and outpatient (**B**) care. Values are adjusted for inflation using the 2020 consumer price index for healthcare and converted to USD using annual exchange rates.

**Table 1:** The baseline characteristics between surgical and matched controls at time zero.

Variables	Surgical Cohort (*n* = 351)		Matched Controls (*n* = 984)		SMD	*p*-Value
	*n*	%	*n*	%		
Age					0.08	0.1620
mean, SD	71.6	6.0	71.0	6.6		
Sex					0.09	0.1626
Male	44	12.5	155	15.8		
Female	307	87.5	829	84.3		
Insurance					0.24	0.0039
NHI	347	98.9	932	94.7		
Medical benefit	3	0.9	36	3.7		
Other	1	0.3	16	1.6		
OA duration					0.24	<0.0001
mean, SD	7.5	14.7	4.4	11.5		
CCI					0.11	0.0536
mean, SD	2.3	2.5	2.6	2.6		
CKD					0.04	0.4981
None	319	90.9	906	92.1		
Yes	32	9.1	78	7.9		
Dementia					0.08	0.2242
None	340	96.9	937	95.2		
Yes	11	3.1	47	4.8		
COPD					0.07	0.3119
None	288	82.1	782	79.5		
Yes	63	18.0	202	20.5		

Note. SMD = standardized mean difference; SD = standard deviation; NHI = National Health Insurance; OA = osteoarthritis; CCI = Charlson comorbidities index; CKD = chronic kidney disease; COPD = chronic obstructive pulmonary disease.

**Table 2:** Medical utilization in the surgical and matched controls at 30-day intervals before and after time zero.

Time Point ^a^	Inpatient Visits		Average LOS		Outpatient Visits	
	Surgical Cohort	Matched Controls	Surgical Cohort	Matched Controls	Surgical Cohort	Matched Controls
	Mean ± SD	Mean ± SD	Mean ± SD	Mean ± SD	Mean ± SD	Mean ± SD
−36	0.07 ± 0.30	0.07 ± 0.28	0.35 ± 1.83	0.33 ± 1.68	1.73 ± 1.51	1.72 ± 1.46
−24	0.03 ± 0.17	0.06 ± 0.27	0.24 ± 1.66	0.21 ± 1.10	1.89 ± 1.96	1.81 ± 1.78
−12	0.11 ± 0.38	0.06 ± 0.28	0.47 ± 1.94	0.15 ± 0.92	1.71 ± 1.50	1.79 ± 1.75
0	1.03 ± 0.20	0.16 ± 0.40	5.17 ± 2.64	0.62 ± 2.40	0.98 ± 1.42	2.29 ± 2.00
12	0.09 ± 0.40	0.06 ± 0.25	0.32 ± 2.10	0.19 ± 1.10	1.56 ± 1.31	1.87 ± 1.88
24	0.06 ± 0.26	0.07 ± 0.30	0.20 ± 1.41	0.28 ± 1.86	1.57 ± 1.35	2.00 ± 1.96
36	0.10 ± 0.37	0.06 ± 0.26	0.42 ± 2.03	0.23 ± 2.36	1.67 ± 1.85	1.91 ± 1.93

Note. SD = standard deviation; LOS = Length of Stay. ^a^ Time points are expressed as 30-day intervals before and after time zero.

**Table 3:** Total medical expenditures in the surgical and matched controls at 30-day intervals before and after time zero.

Time Point ^a^	Inpatient		Outpatient	
	Surgical Cohort	Matched Controls	Surgical Cohort	Matched Controls
	Mean ± SD	Mean ± SD	Mean ± SD	Mean ± SD
−36	245.1 ± 1527.1	221.1 ± 1270.7	152.1 ± 276.5	160.1 ± 324.3
−24	329.1 ± 2840.1	134.9 ± 830.6	156.8 ± 250.6	170.7 ± 516.8
−12	182.8 ± 727.1	107.8 ± 748.0	114.4 ± 181.5	138.0 ± 281.7
0	7102.2 ± 2675.4	526.0 ± 2336.3	41.9 ± 107.0	131.3 ± 210.0
12	213.2 ± 1153.4	126.4 ± 1144.5	106.1 ± 123.1	146.5 ± 258.8
24	127.0 ± 760.3	223.4 ± 1793.0	111.8 ± 144.3	172.2 ± 304.3
36	348.9 ± 1737.6	166.5 ± 1288.8	101.8 ± 124.9	162.0 ± 260.4

Note. SD = standard deviation. Medical expenditures were adjusted for inflation using the 2020 healthcare consumer price index and converted to US dollars based on annual exchange rates. ^a^ Time points are expressed as 30-day intervals before and after time zero.

**Table 4:** The GEE regression results for medical utilization and expenditure measures, before and after surgery.

	Before Surgery		After Surgery	
Dependent Variables	exp(β) (95% CI) ^b^	*p*-Value ^d^	exp(β) (95% CI) ^c^	*p*-Value ^d^
Utilization ^a^				
Inpatient visits	0.73 (0.62–0.87)	0.0003	2.09 (1.64–2.67)	<0.0001
LOS	0.88 (0.70–1.10)	0.2544	2.49 (2.02–3.07)	<0.0001
Outpatient visits	1.05 (0.95–1.17)	0.3514	0.74 (0.65–0.84)	<0.0001
Expenditures				
Inpatient				
Covered	0.75 (0.65–0.86)	<0.0001	3.12 (2.51–3.89)	<0.0001
Uncovered	0.76 (0.67–0.87)	<0.0001	2.93 (2.41–3.58)	<0.0001
OOPs	0.75 (0.65–0.86)	<0.0001	2.99 (2.42–3.71)	<0.0001
Outpatient				
Covered	1.19 (0.96–1.48)	0.1144	0.52 (0.38–0.70)	<0.0001
Uncovered	1.08 (0.81–1.45)	0.5904	0.37 (0.24–0.58)	<0.0001
OOPs	1.46 (1.30–1.64)	<0.0001	0.57 (0.48–0.68)	<0.0001

Note. LOS = length of stay; OOPs = out-of-pocket payments; CI = Confidence Interval. ^a^ Poisson models were applied for inpatient and outpatient visits; Negative Binomial was applied for LOS, due to overdispersion and excess zeros. ^b^ Adjusted for time point, age, sex, insurance type, degenerative osteoarthritis, duration of degenerative osteoarthritis, TKA re-surgery, CCI, CKD, dementia, COPD, and year fixed effects. ^c^ Estimated as the sum of the surgical cohort and post-surgery indicator coefficients, with standard errors derived from the model’s covariance matrix. ^d^ Bonferroni-corrected significance threshold (18 estimates): α ≈ 0.0028.

## Data Availability

The data used in this study are owned by the hospital and are not publicly available due to institutional and patient privacy restrictions, as they were derived from hospital claims records. The data may be available from the corresponding author upon reasonable request and with permission from the hospital.

## References

[1] Cross M., Smith E., Hoy D., Nolte S., Ackerman I., Fransen M., Bridgett L., Williams S., Guillemin F., Hill C.L. (2014). The global burden of hip and knee osteoarthritis: Estimates from the global burden of disease 2010 study. Ann. Rheum. Dis..

[2] Steinmetz J.D., Culbreth G.T., Haile L.M., Rafferty Q., Lo J., Fukutaki K.G., Cruz J.A., Smith A.E., Vollset S.E., Brooks P.M. (2023). Global, regional, and national burden of osteoarthritis, 1990–2020 and projections to 2050: A systematic analysis for the Global Burden of Disease Study 2021. Lancet Rheumatol..

[3] Ministry of the Interior and Safety (2026). Resident Registration Population Status: Population by Administrative District and 5-Year Age Group. https://kosis.kr/statHtml/statHtml.do?orgId=101&tblId=DT_1B04005N&conn_path=I2.

[4] Hügle T., Geurts J., Nüesch C., Müller-Gerbl M., Valderrabano V. (2012). Aging and Osteoarthritis: An Inevitable Encounter?. J. Aging Res..

[5] Li Y., Wei X., Zhou J., Wei L. (2013). The Age-Related Changes in Cartilage and Osteoarthritis. BioMed Res. Int..

[6] Neuprez A., Neuprez A.H., Kaux J.-F., Kurth W., Daniel C., Thirion T., Huskin J.-P., Gillet P., Bruyère O., Reginster J.-Y. (2020). Total joint replacement improves pain, functional quality of life, and health utilities in patients with late-stage knee and hip osteoarthritis for up to 5 years. Clin. Rheumatol..

[7] Wilson R.A., Gwynne-Jones D.P., Sullivan T.A., Abbott J.H. (2021). Total hip and knee arthroplasties are highly cost-effective procedures: The importance of duration of follow-up. J. Arthroplast..

[8] Qadeer A.S., Meher A., Rachel J., Paulson W., Patra A., Gandhi N., Ay N., Nanda L., Rout S.K., Dutta A. (2025). Economic Evaluation of Total Knee Replacement Compared with Non-Surgical Management for Knee Osteoarthritis in India. PharmacoEconomics-Open.

[9] Mota R.E.M., Tarricone R., Ciani O., Bridges J.F.P., Drummond M. (2012). Determinants of demand for total hip and knee arthroplasty: A systematic literature review. BMC Health Serv. Res..

[10] Merle-Vincent F., Couris C.M., Schott A.-M., Conrozier T., Piperno M., Mathieu P., Vignon É. (2011). Factors predicting patient satisfaction 2 years after total knee arthroplasty for osteoarthritis. Jt. Bone Spine.

[11] Aynardi M., Post Z., Ong A., Orozco F., Sukin D.C. (2014). Outpatient surgery as a means of cost reduction in total hip arthroplasty: A case-control study. HSS J. Musculoskelet. J. Hosp. Spec. Surg..

[12] Pasqualini I., Turan O., Emara A.K., Ibaseta A., Xu J., Chiu A., Piuzzi N.S. (2024). Outpatient total hip arthroplasty volume up nearly 8-fold after regulatory changes with expanding demographics and unchanging outcomes: A 10-year analysis. J. Arthroplast..

[13] Singh J.A., Yu S., Chen L., Cleveland J.D. (2019). Rates of total joint replacement in the United States: Future projections to 2020–2040 using the national inpatient sample. J. Rheumatol..

[14] Culliford D., Maskell J., Judge A., Cooper C., Prieto-Alhambra D., Arden N.K. (2015). Future projections of total hip and knee arthroplasty in the UK: Results from the UK Clinical Practice Research Datalink. Osteoarthr. Cartil..

[15] Kim Y.-B., Choi H.-S., Kang E.M., Park S., Seo G.-W., Chun D.-I., Min T.-H. (2021). Trends of total knee arthroplasty according to age structural changes in Korea from 2011 to 2018. Int. J. Environ. Res. Public Health.

[16] Ron D., Daley A.B., Coe M.P., Herrick M.D., Roth R.H., Abess A.T., Martinez-Camblor P., Deiner S.G., Boone M.D. (2025). Frailty and associated healthcare expenditures among patients undergoing total hip and knee arthroplasty. J. Frailty Aging.

[17] Hancock J.U., D’Alonzo J.A., Simon P., Gill M.S., Bernasek T.L., Lyons S.T. (2025). Arthroplasty in Medicare: A Cost Distribution Analysis of Medicare Beneficiaries Undergoing Total Knee Arthroplasty. J. Arthroplast..

[18] Maman D., Laver L., Becker R., Mahamid A., Berkovich Y. (2025). Robotic-assisted total knee arthroplasty reduces postoperative complications and length of stay without increased cost compared to navigation-guided techniques: A national analysis. Knee Surg. Sports Traumatol. Arthrosc..

[19] Magruder M.L., Yao V.J., Rodriguez A.N., Ng M.K., Sasson V., Erez O. (2023). Does semaglutide use decrease complications and costs following total knee arthroplasty?. J. Arthroplast..

[20] Molloy I.B., Martin B.I., Moschetti W.E., Jevsevar D.S. (2017). Effects of the length of stay on the cost of total knee and total hip arthroplasty from 2002 to 2013. J. Bone Jt. Surg..

[21] Kim S.J., Park E.-C., Jang S.I., Lee M., Kim T.H. (2013). An analysis of the inpatient charge and length of stay for patients with joint diseases in Korea: Specialty versus small general hospitals. Health Policy.

[22] Ministry of Data and Statistics (2025). Consumer Price Index Survey: Consumer Price Index by Expenditure Purpose. https://kosis.kr/statHtml/statHtml.do?orgId=101&tblId=DT_1J22001&conn_path=I2.

[23] Ministry of Economy and Finance (2025). Survey on Exchange Rates by Currency. https://www.index.go.kr/unity/potal/main/EachDtlPageDetail.do?idx_cd=1068.

[24] Glasheen W.P., Cordier T., Gumpina R., Haugh G., Davis J., Renda A. (2019). Charlson comorbidity index: ICD-9 update and ICD-10 translation. Am. Health Drug Benefits.

[25] Suh C.Y., Lee Y.J., Shin J.-S., Lee J., Kim M.-r., Koh W., Cha Y.-Y., Shin B.-C., Hwang E.-H., Suhr K. (2017). Analysis of medical service use of knee osteoarthritis and knee meniscal and ligament injuries in Korea: A cross-sectional study of national patient sample data. BMC Musculoskelet. Disord..

[26] Bozic K.J., Stacey B., Berger A., Sadosky A., Oster G. (2012). Resource utilization and costs before and after total joint arthroplasty. BMC Health Serv. Res..

[27] Kohring A.S., Lutz R., Parikh N., Hobbs J., Bridges T.N., Krueger C.A. (2025). Analysis of Costs Associated with Increased Length of Stay After Total Joint Arthroplasty at a Single Private Practice. JAAOS—J. Am. Acad. Orthop. Surg..

[28] Lee J.-H., Kim H.-J. (2019). Identification of spatial distribution of an aged population and analysis on characterization of the cluster: Focusing on Seoul Metropolitan Area. J. Digit. Contents Soc..

[29] Jeon B., Jung B., Song Y.-K. (2026). Post-discharge healthcare utilization and costs in musculoskeletal surgery patients: A cohort study in Korea. PLoS ONE.

[30] Xue L., Yan Y., Chen Z., Hong Y. (2025). Acupuncture and massage therapy enhance postoperative recovery and quality of life in orthopedic patients. Medicine.

[31] Cho W., Shin W.-C., Kim H., Chung W.-S., Song M.-Y., Youn Y., Cho J.-H. (2025). Acupuncture for post-operative pain relief and functional improvement in tibial fracture: A systematic review and meta-analysis. Healthcare.

[32] Ga H. (2024). The Korean Long-Term Care System: 2024 Update. Ann. Geriatr. Med. Res..

[33] Tschon M., Contartese D., Pagani S., Borsari V., Fini M. (2021). Gender and Sex Are Key Determinants in Osteoarthritis Not Only Confounding Variables. A Systematic Review of Clinical Data. J. Clin. Med..

[34] Ripollone J.E., Huybrechts K.F., Rothman K.J., Ferguson R.E., Franklin J.M. (2020). Evaluating the Utility of Coarsened Exact Matching for Pharmacoepidemiology Using Real and Simulated Claims Data. Am. J. Epidemiol..

[35] Nguyen T.-L., Collins G.S., Spence J., Daurès J.-P., Devereaux P., Landais P., Le Manach Y. (2017). Double-adjustment in propensity score matching analysis: Choosing a threshold for considering residual imbalance. BMC Med. Res. Methodol..

